# Quantum ESPRESSO: One Further Step toward the Exascale

**DOI:** 10.1021/acs.jctc.3c00249

**Published:** 2023-07-31

**Authors:** Ivan Carnimeo, Fabio Affinito, Stefano Baroni, Oscar Baseggio, Laura Bellentani, Riccardo Bertossa, Pietro Davide Delugas, Fabrizio Ferrari Ruffino, Sergio Orlandini, Filippo Spiga, Paolo Giannozzi

**Affiliations:** † SISSA, Scuola Internazionale Superiore di Studi Avanzati, via Bonomea 265, 34136 Trieste, Italy; ‡ CINECA, Via Magnanelli 6/3, 40033 Casalecchio di Reno, BO, Italy; ¶ CNR-IOM Istituto dell’Officina dei Materiali, area SISSA, via Bonomea 265, 34136 Trieste, Italy; § CINECA, Via dei Tizi 6/b, 00185 Roma, RM, Italy; ∥ NVIDIA Corporation, 2788 San Tomas Expressway, Santa Clara, California 95051, United States; ⊥ Dipartimento di Scienze Matematiche, Informatiche e Fisiche (DMIF), Università degli Studi di Udine, via delle Scienze 206, 33100 Udine, Italy

## Abstract

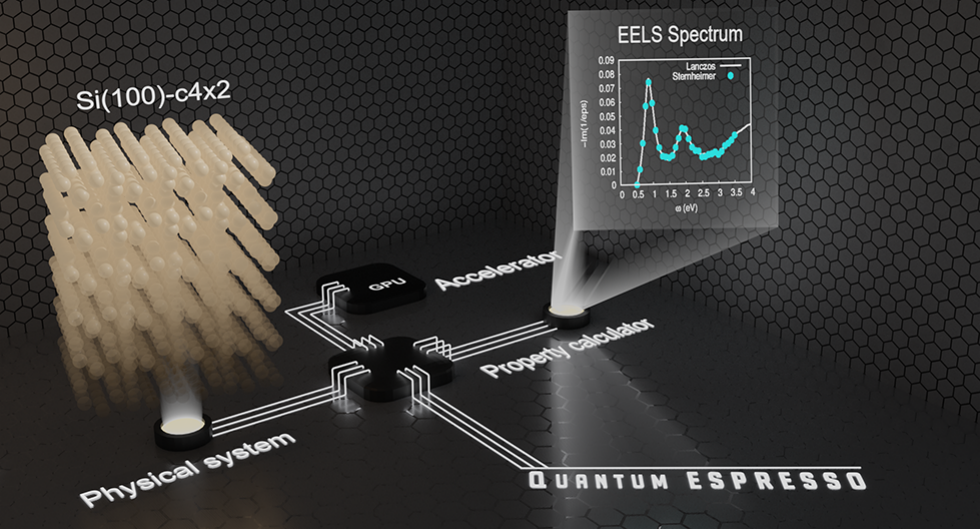

We review the status of the Quantum ESPRESSO software suite for electronic-structure calculations based on plane waves, pseudopotentials, and density-functional theory. We highlight the recent developments in the porting to GPUs of the main codes, using an approach based on OpenACC and CUDA Fortran offloading. We describe, in particular, the results achieved on linear-response codes, which are one of the distinctive features of the Quantum ESPRESSO suite. We also present extensive performance benchmarks on different GPU-accelerated architectures for the main codes of the suite.

## 1 Introduction

High-performance computing (HPC) is approaching the *exascale*, that is, 10^18^ floating-point operations per seconds (flops). This means that calculations that took hundreds of hours 30 years ago could now be performed in tens of seconds, at least in principle. HPC has thus become a strategic asset for industrial and technological development of countries. Several exascale and pre-exascale machines are in the preproduction state or already fully operational. For such a class of machines, graphics processing unit (GPU) acceleration has become a *de facto* standard, and almost all of the first five entries in the Top500^1^ list of supercomputers are currently based on GPU acceleration.

This context has proved to be particularly fertile for molecular and material sciences that have evolved in parallel with the advances in computer science. Nowadays, most of the main codes for molecular and material modeling are accelerated or are in the process of being ported to accelerated architectures. In this respect, the Quantum ESPRESSO software suite^2−5^ can boast a long experience: the first accelerated working version dates back to several years ago (2017), and the release qe-6.4 (March 2019) was the first one to be officially distributed by the Quantum ESPRESSO Foundation(6) having a GPU counterpart for the most important core functionalities. This first porting phase, covering only the main self-consistent code PWscf, is described in ref (2).

Since then, a great effort has been devoted to the improvement of the GPU version of PWscf and to the porting of the other codes of the suite to GPU-accelerated architectures. The latest release of Quantum ESPRESSO, namely, qe-7.2, enables GPU execution of the linear-response codes: PHonon,^7,8^ turboEELS,^9,10^ turboLanczos,^11,12^ HP,^13−15^ and of the molecular-dynamics code CP.^16^

The aim of this work is thus dual: on the one hand, we want to disclose some important developments done in the GPU porting of Quantum ESPRESSO since the first article^2^ was published. On the other hand, we also want to provide more detailed information—that is missing in the literature—about performances of the codes of the suite on the current state-of-art HPC supercomputers, highlighting advantages, drawbacks, and the most effective parallelization schemes for GPU execution.

The structure of this paper is as follows: Section 2 describes the main new developments of the codes, together with the general philosophy and the technical approach followed for the porting; Section 3 presents selected benchmark tests on GPU for some of the main codes (PWscf, PHonon, turboEELS, CP) of the Quantum ESPRESSO suite; Section 4 contains our conclusions.

## 2 Code Development

### 2.1 General Philosophy

Our approach has been developed within the *separation of concerns*(2) philosophy: ideally, method developers in science departments and research laboratories should be concerned with the calculation of physical properties, disregarding architectural details, whereas scientists and research engineers in IT departments and HPC centers should focus on low-level mathematical and system libraries. Separation of concerns is the overarching guideline for the action of the EU MaX Centre of Excellence (CoE) for HPC Applications,^17^ whose mission is to foster the porting of important community codes for quantum materials modeling to heterogeneous architectures. The MaX way to separation of concerns is to refactor community codes into a software stack of conceptually distinct—though in practice partially overlapping—components. The core of the code is a *quantum engine* whose main purpose is to perform *Hamiltonian builds*, i.e., the application of the Hamiltonian operator to molecular/Bloch orbitals and related operations, and to solve the quantum-mechanical equations that determine them and their response to external perturbations. The quantum engine is complemented by various *property calculators*, designed to evaluate specific properties and to simulate specific processes of molecular and extended systems. Both the quantum engine and the property calculators leverage a number of modules and mathematical and system libraries. Modules are homogeneous software components that share the same coding style and naming conventions and may share global variables with other modules, with the quantum engine and property calculators. Modules are not designed for extended portability and their adoption in third-party software in general requires the adoption of at least some of the internal data structures of Quantum ESPRESSO. Domain-specific mathematical libraries address various general-purpose mathematical operations (e.g., three-dimensional fast Fourier transforms, linear algebra based on both factorization and iterative techniques, minimization and extrapolation to self-consistency, etc.). Ideally, libraries should not rely on any global variables but trade data with the calling program units only through well-designed public application programming interfaces (APIs). Although domain-specific libraries are specialized for and take advantage of the specific features of plane-wave electronic-structure codes, they can be easily adopted by third-party codes without major concerns about their internal data structure. Finally, system libraries perform various low-level tasks, including the offload of data to hardware accelerators, the management of inter- and intranode data communications, etc. System libraries play a key role in our sustainable model of software development and maintenance. By abstracting as much as possible data management and communications, it is possible to maintain a large code base largely independently of the underlying hardware architecture, thus avoiding or dramatically limiting code duplication and freeing the developers of high-level software layers from the need to operate with hardware-specific directives.^2^

### 2.2 GPU Porting

**Figure 1:**
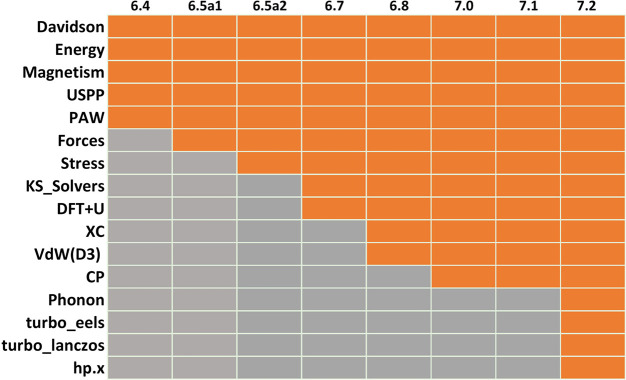
Progression of GPU porting over the different versions of the Quantum ESPRESSO suite. In gray and orange are the nonported and ported features, respectively.

In Figure 1 a brief summary of the progress of the porting of the Quantum ESPRESSO suite over subsequent releases is shown, starting from release qe-6.4 (March, 2019) to the last qe-7.2 version (March 2023).

**Figure 2:**
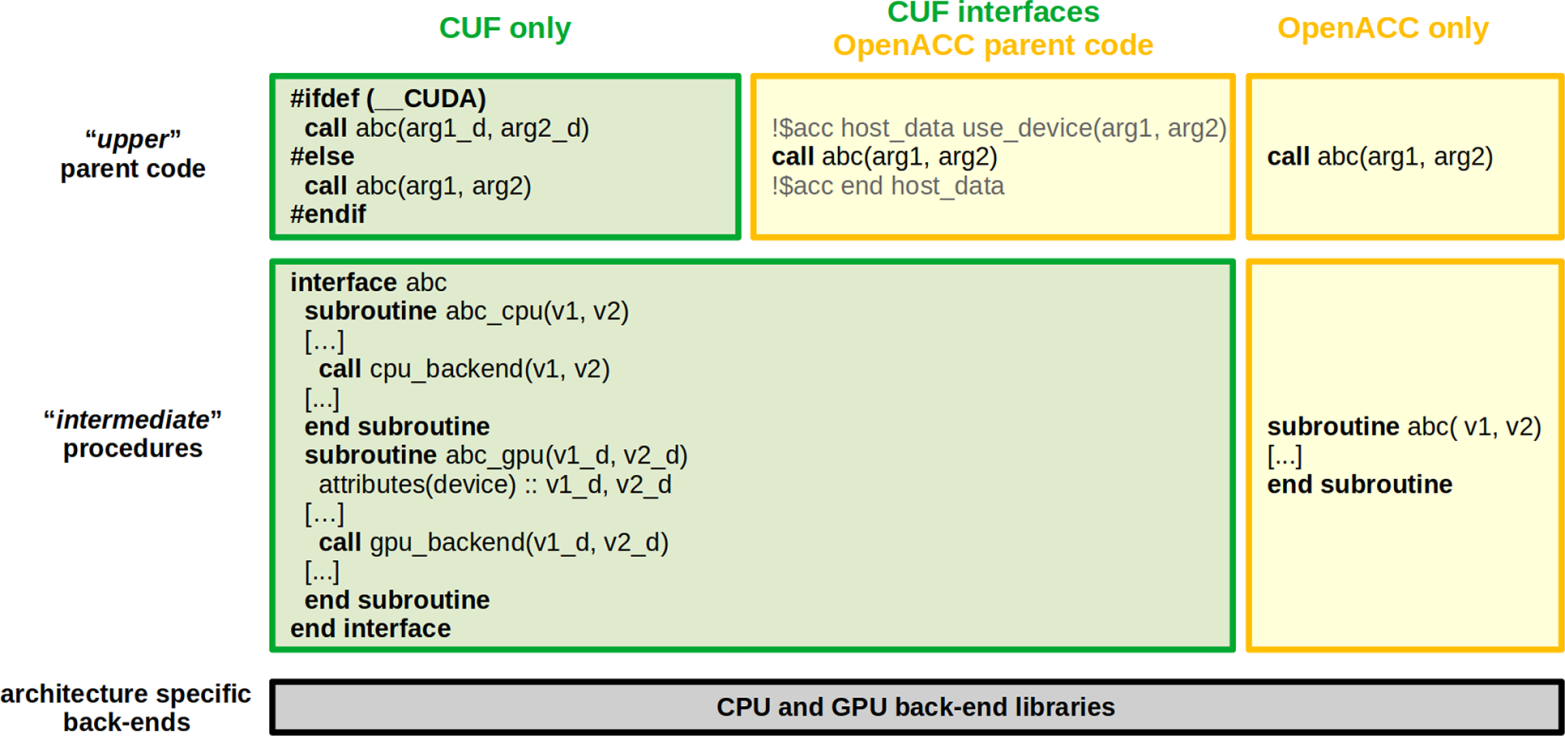
Comparison between CUDA Fortran and OpenACC codes.

Earlier versions of the suite were accelerated using a programming model fully based on the CUDA Fortran language, that, on the one hand, provided significant speed-ups with respect to the nonaccelerated counterpart but, on the other hand, intrinsically required duplication of code and variables, on host and device sides (see, for example, the pseudocode in Figure 2). As a consequence, for each accelerated portion of the native Fortran code, a number of “_gpu.f90” files were created that included GPU counterparts of the original subroutines and modules.

**Figure 3:**
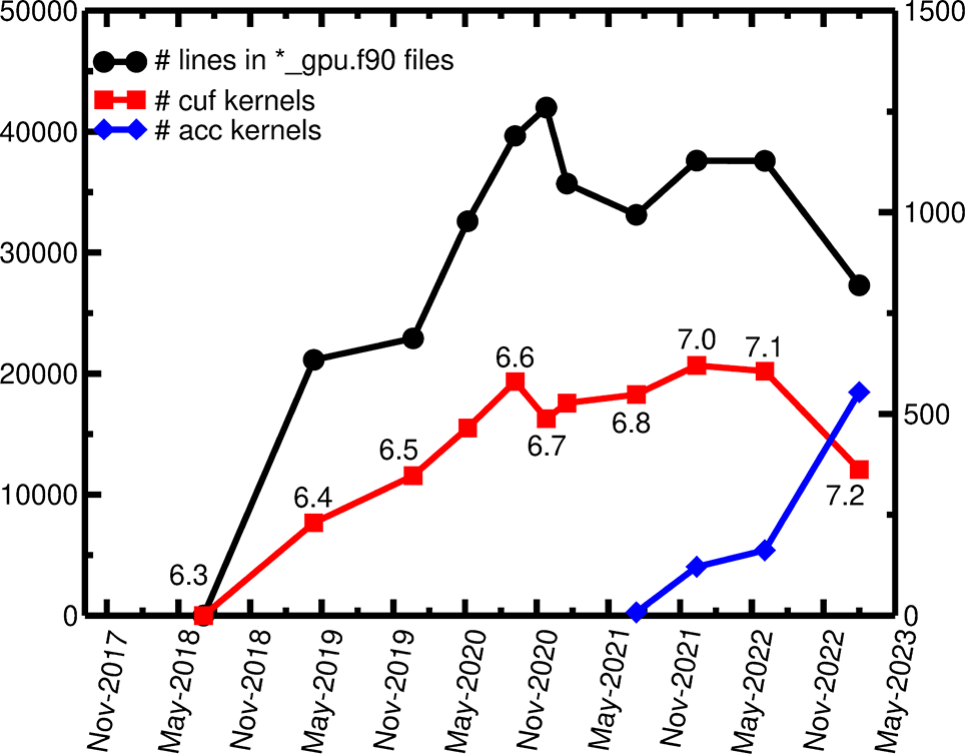
Estimate of CUDA Fortran and OpenACC directives in the Quantum ESPRESSO codes over the years, where the reference release version has been specified along the red line. Black line refers to the left *y*-axis, red and blue lines refer to the right *y*-axis, and “acc kernels” includes both OpenACC kernels and parallel directives.

Figure 3 shows a steady increase in the number of lines of “_gpu.f90” files with an increasing number of CUDA Fortran (”cuf”) kernels, until the end of 2020. As the number and size of the “_gpu.f90” files increased, the maintenance burden also increased accordingly, hampering the porting of new features. Furthermore, as mentioned in the previous section, Quantum ESPRESSO is a community code where people with different backgrounds are encouraged to contribute. Since developers with no or little experience on GPU programming tend to add features only to the CPU pathway of the codes, the two pathways easily tend to diverge, and it becomes very difficult to keep them aligned in the long term and to identify and fix bugs.

For these reasons, we switched to an alternative porting model, mainly based on OpenACC, which also exploits OpenACC/CUDA Fortran interoperability. The main advantage of this approach, as briefly shown in Figure 2, is that in this case host and device copies of the variables are managed by directives and are referenced as the same variable in the parent code, thus allowing us to have a unique source code for both CPU and GPU compilation and execution. In this way, the general structure of the code tends to remain consistent even in the case of features added only on the CPU side. Notably, CUDA Fortran has not been completely removed but it has been retained in those cases for which it led to a clear advantage. For example, it can be useful when the differences between CPU and GPU architectures can be better exploited using different algorithms, e.g., in case of large loops batched on the cache size or Fast Fourier Transforms (FFT), where in the CPU execution one band at time is processed, whereas the heavy internal parallelism of GPU encourages us to process many bands at once. Another example is for Fortran interfaces (see Figure 2), where the “device” attribute allows us to trigger specific host or device procedures (there is not an equivalent within the OpenACC framework). The latter case is especially helpful when general libraries call system-specific backends of numerical libraries, which are specialized for host or device execution. For example, the *abc* interface referenced in Figure 2 can be representative of FFTXlib or LAXlib, which internally call different host or device specific numerical backends (e.g., cuFFT or cuSOLVER, referenced as *cpu_backend* and *gpu_backend*) to respectively perform FFTs and solve eigenproblems. Also, inside UtilXlib, different MPI backends can be linked. OpenACC/CUDA Fortran interoperability has also been exploited to progressively substitute CUDA Fortran portions with OpenACC directives, avoiding the rewriting of an entire new code from scratch. After the adoption of this approach in release qe-6.8 (beginning 2021) the number of lines of code contained in the duplicated “_gpu.f90” files dropped down as the number of OpenACC directives increased, even with an increased number of ported features (compare Figures 3 and 1). For example, in the latest qe-7.2 release, the linear-response codes (PHonon, turboEELS) are ported to GPU, but the number of lines in the “_gpu.f90” files decreased by more than 10000 with respect to the previous qe-7.1 release.

After this refactoring, the code is more clearly separated into conceptually different layers with different scopes. In Figure 2 an “upper” layer of code can be schematically identified, which contains most of the physics of the calculation, is strongly based on OpenACC directives, and is quite agnostic of the underlying computational architecture. Developers with no or little experience in GPU programming can easily continue to work here. A lower layer of code (represented by the *abc* interface and subroutine) is based on more specific procedures that specialize the computation for CPU and/or GPU architectures. These procedures can be easily called in a simple way from the parent code. Internally, these procedures further specialize execution, calling numerical specific libraries (represented by *cpu_backend* and *gpu_backend*) that are used as the final backends of the computation and whose development is the domain of computer scientists and deeply specialized programmers. This is in line with the aforementioned separation of concerns philosophy,^2,17^ described in the previous section.

### 2.3 Eigensolver

The default GPU implementation^2^ of the eigenvalue solver is based on the cuSOLVER library: real and complex Hamiltonian matrices are diagonalized with the generalized eigenvalue problems using a single GPU. Although very fast, this approach is limited by the available GPU memory, becoming problematic for very large matrices (e.g., dimension beyond ∼7000 on a V100 NVIDIA GPU with 16GB VRAM). Moreover, the computational cost of a dense eigenproblem solution scales as  and may become sizable for large matrices.

A recently implemented alternative approach (not yet available in the production version) of a parallel dense-matrix eigensolver on GPU is based on the Eigenvalue soLvers for Petascale Applications (ELPA)^18^ library. ELPA implements two types of distributed eigensolvers: one-stage (ELPA1) and two-stage (ELPA2) diagonalization methods. The latter algorithm is the most suitable for distributed-memory architectures because it scales more efficiently, as global communications are not required, and it has a faster computational part.

The ELPA library relies on the matrix layout defined by the ScaLAPACK library for parallel linear algebra; thus, it can be used as a drop-in improvement in ScaLAPACK-based applications like Quantum ESPRESSO.

### 2.4 Exchange–Correlation Library

Starting from the qe-6.8 version, the code for computing exchange–correlation (XC) kernels of Quantum ESPRESSO was encapsulated into an independent library that also supports the usage of other external XC libraries in a flexible way. The aim of this refactoring was twofold: to ease the maintenance and the development (notably, the addition of new functionals) of the XC code and to seamlessly integrate the functionalities of the popular Libxc library^19^ into Quantum ESPRESSO. The XC library can be used by other electronic-structure softwares as well.

The library covers the local-density (LDA), generalized-gradient (GGA), and meta-GGA families of XC functionals. It is interfaced to Quantum ESPRESSO through a set of wrapper routines, which call either the internally provided functionals or the ones from Libxc, depending on the input choice. Therefore, the library consists of a few main routines that provide the energy, potential, and potential derivatives on the density grid and a number of initialization and setting routines that manage additional dependencies. The library allows any combination of internal and external (Libxc) functional forms.

The computational cost of the XC library is typically a small fraction of the total. Nonetheless a GPU porting allows one to avoid data movement and to significantly improve the performance in the exchange–correlation potential calculation. The simplicity of the driving algorithm: one main loop running over the density grid, where the routines computing the functional are called at each point, ensures optimal speed-up with little intervention (a few OpenACC directives) on the code. The input density (and possibly its derivatives) and the output energy and potential arrays of the main XC routines are assumed to be present on device memory, depending upon the value of a logical optional variable. If the latter is false (or omitted), then the offload is done internally to the library so that the developer is not forced to care about the offloading.

### 2.5 Hybrid Functionals

The computation of the Fock operator for hybrid functionals^20−22^ is still a hard task for plane-wave-based codes. In this respect, a couple of major advances have been included in the Quantum ESPRESSO suite in the last years. In 2017, a new scheme for the parallel computation of exact exchange based on a band-pair parallelization approach was proposed by Barnes et al.^23^ and integrated in the Quantum ESPRESSO suite. More or less in the same period, Lin developed a new method based on an Adaptively Compressed Exchange (ACE) operator^24,25^ that allowed us to tear down the computational time of the self-consistent field (SCF) step with no loss of accuracy. Such a method was then implemented^26^ in the Quantum ESPRESSO suite with some minor modifications, together with a variant that exploits orbital localization^27,28^ to further reduce the computational burden. Benchmark tests showed a dramatic decrease in computational time to solution with respect to previous implementations. On top of these methodological developments, the entire exact-exchange code has been ported to GPU.^2^

Recently, the implementation of hybrid functionals in Quantum ESPRESSO has been improved with some minor changes. Every time the ACE projector is updated during an SCF calculation, it is also written on disk, in the same format used for wave functions I/O, so that it can be read to speed-up subsequent runs. The overall amount of memory and I/O bandwidth required for storing the ACE projector is quite relevant but generally affordable, as it is comparable to the memory needed for storing the wave functions. This feature is particularly useful in case one large calculation with an hybrid functional is stopped or crashes before reaching convergence, for example, due to wall time limit policies of the queue systems of HPC centers. In this case, the calculation can be recovered by reading the ACE projector and the wave functions from disk and restarting the calculation from the last (previously stopped or crashed) outer iteration, skipping the first exact exchange calculation of the restart.

The feature mentioned above is also very useful for band-structure calculations. In this case, one usually needs to perform a very heavy SCF calculation on a large and dense uniform (Monkhorst–Pack^29^) grid, including many virtual orbitals to compute unoccupied band energies. Then one resorts to some interpolation scheme, e.g., using Wannier functions,^30−32^ to compute the band structure for the desired **k**-points. With the new feature, the heavy SCF can be split into two steps: a cheaper SCF run on occupied bands only and a subsequent non-SCF run including also virtual orbitals, thus avoiding the evaluation of the ACE operator on the virtual manifold at each SCF outer iteration. Noteworthy, since the virtual orbitals are not included in the first SCF step, in the non-SCF procedure the ACE potential is first read from file and used as is for a first diagonalization; then it is updated with the new virtual orbitals and a second diagonalization is performed to get correct virtual band energies. Currently, it is not yet possible to perform the non-SCF calculation on a set of **k**-points different from the one used in the previous SCF run.

As a last remark, a computationally inexpensive method^33,34^ has been recently implemented in order to interpolate band structures directly from SCF or non-SCF calculations on uniform Monkhorst–Pack grids. This method is based on a fitting algorithm that minimizes a roughness function across the entire Brillouin zone and can be used as a quick alternative to using the Wannier functions.^35−37^

### 2.6 Parallelism and Data Distribution

Different levels of parallelism, based on both the message-passing interface (MPI) library and on multithreading, are currently implemented in the Quantum ESPRESSO suite. They can be flexibly combined together on the basis of the particular molecular system under study and the hardware architecture. In the following, the different parallelization schemes are briefly summarized with a focus on accelerated architectures, in order to facilitate the discussion of the performance on GPU in Section 3. More details about parallelism in Quantum ESPRESSO can be found in the original works^2−5^ and other more tailored publications.^38^

**Figure 4:**
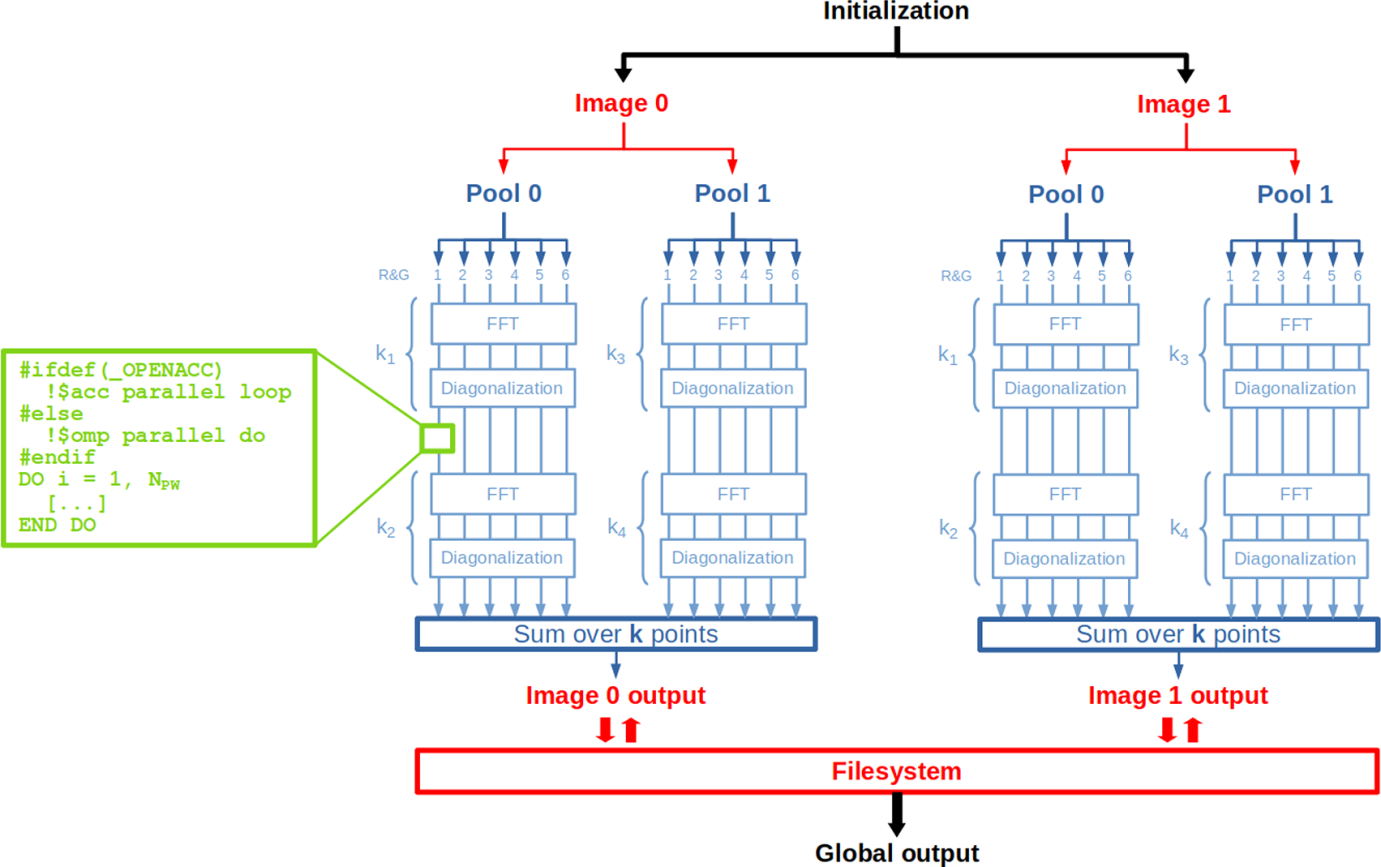
Sample execution flow with 2 *images* and four **k**-points, distributed in 2 *pools* and 24 total ranks. Global operations are represented in black, operations involving *images*, *pools*, and *R&G* groups are represented in red, dark blue, and light blue, respectively, and GPU acceleration and OpenMP multithreading are in the green box. Closed boxes represent operations that require communications among *images* (Filesystem access), *pools* (sum over **k**-points), and *R&G* groups (FFT, diagonalization).

The diagram in Figure 4 shows in a simplified way how the main types of parallelism implemented in the Quantum ESPRESSO codes are interconnected with each other in a prototypical execution. The outermost level of parallelism is the *image* parallelism, whereby different MPI ranks run different instances of a given calculation, and the communications are mostly performed through filesystem I/O. This scheme is very useful for algorithms that expose a natural parallelism based on repeated clearly distinguishable tasks that need to communicate with each other only to some very small extent. This is the case, for example, of Nudged Elastic Band (NEB)^39−41^ calculations, where each *image* computes an independent geometry along the transition pathway. Geometries are connected by springs, and each *image* has to communicate only forces to the others. Another example is phonon calculations, where different *images* can concurrently solve different sets of Sternheimer equations^42^ for different irreducible representations of nuclear displacements and/or for different wavevectors **q**.

Other levels of parallelism are *pools*, *band groups*, and *plane-wave* schemes (the latter also often referred to as *R&G*), that hierarchically distribute memory and computational load of those parts of the code that depend on the number of **k**-points (*N*_*k*_), bands (*N*_*b*_) and plane waves (*N*_PW_), respectively.

*Pool* parallelism can be used for all systems described by more than one **k**-point and/or nondegenerate spin channels. In this case, the total number of MPI ranks available for the calculation, for example, *N*_tot_, is divided into *N*_pools_ groups (*pools*) of *N*_tot_/*N*_pools_ ranks each. *N*_pools_ distinct *R&G* data structures are allocated and distributed among their respective *pools*’ groups and operate over separated sets of preassigned **k**-points. The efficiency of this type of parallelism—which is similarly exploited in many quantum-chemistry codes—relies on the fact that the communications among the *pools* are limited because the code performs most of the operations autonomously within each eigenspace of the translational symmetry group. For example, in Figure 4 communications among *pools* are graphically represented by summations over **k**-points only at the end of the execution and are not required by FFT and diagonalization steps. A typical example in the Quantum ESPRESSO suite is the Kohn-Sham solver (KS_Solver) library, where the Kohn–Sham equations are solved independently for each **k**-point and most of the communications among *pools* are needed only once the Kohn–Sham orbitals are found, to obtain the total electronic energy and density. In this respect, recalling the example in Figure 4, one particularly efficient setup is when all ranks belonging to the same *pool* are executed on the same node (e.g., one *pool* per node, two *pools* per node, etc.), because internode communications are avoided in FFT and diagonalization steps. Furthermore, concerning GPU calculations, since each GPU is internally massively parallel (e.g., the V100 architecture has 5120 CUDA cores, for an overall peak double-precision performance of 7.8 TFLops), in many cases it is feasible to move the whole *R&G* parallelism within one single GPU, removing most of the communication overhead and yielding a significant performance boost. In Figure 4, it is evident that using one rank per *pool* drastically reduces also intranode communications in FFT and diagonalization steps.

*Band group* parallelism works on conceptually similar grounds to the *pool* parallelism, also for systems where the latter cannot be used. The total number of ranks *N*_tot_, or the total number of *N*_tot_/*N*_pools_ ranks inside each *pool*, is further divided into *N*_bg_*band groups*, each composed of *N*_tot_/*N*_pools_/*N*_bg_ MPI ranks.

Finally, the ranks can also be used to distribute the data structures that depend on the number of plane waves (*R&G*). This latter parallelization is very effective in particular from the point of view of memory requirements, as it allows us to distribute memory along the largest dimension of the data structures. For example, in Figure 4, the plane-waves of each *pool* are distributed among 6 ranks.

As a general remark that will be often relevant for the following benchmarks, increasing the number of *pools* when possible is beneficial for performance but requires more memory, whereas decreasing the number of *pools* and increasing the dimension of each *pool* group with *R&G* parallelism is beneficial for memory consumption.

Another important level of parallelism, compatible with all the aforementioned ones, is related to the most important linear algebra operations (most noticeably diagonalization and matrix–matrix product) that are performed using distributed linear algebra libraries, e.g., Scalapack, ELPA.

Finally, OpenMP multithreading (OMP) can be used to accelerate loops at the finest level, inside each rank (see the green box in Figure 4). In case of CPU-only calculations, OMP threads can be effectively exploited especially when many nodes are needed for memory reasons, but deploying all the available CPU cores with MPI ranks leads to communication overheads. The optimal number of OMP threads can vary depending on the particular molecular system size and node configuration. From our experience based on the current HPC architectures, a good choice is usually to associate 2 to 8 OMP-threads per MPI rank.

In the case of GPU-accelerated calculations, a good practice is to bind the number of MPI ranks to the number of available GPUs, so as to work with one MPI rank per GPU. Other choices, involving GPU oversubscription, are usually less convenient. In this case, since usually HPC architectures are provided with more CPU cores than GPUs per node, OMP threads can be used to deploy all of the remaining CPU cores not deployed with MPI ranks. The effect on performance is however small if the GPU porting is effective, as usually only a small fraction of the computational workload is executed on the CPU.

## 3 Code Performance on Selected Benchmark Tests

In this section we report some results on computational performance of the main codes of the Quantum ESPRESSO suite on GPU-accelerated machines, in terms of times, speed-up, and parallel efficiency.

Reference “CPU” and “GPU” calculations have been performed respectively on Galileo100 and Marconi100 clusters at CINECA, whereas for some selected very large systems we also used a third cluster, Selene, made of NVIDIA DGX A100 nodes, each with 8 GPUs. Table 1 briefly summarizes the main technical features of the three clusters.

**Table 1:** System Specification for the GPU (Marconi100 and Selene) and CPU (Galileo100) Partitions

Cluster	Galileo100	Marconi100	Selene
Centre	CINECA	CINECA	NVIDIA Corp
Model	Dual-Socket Dell PowerEdge	IBM Power AC922 (Whiterspoon)	NVIDIA DGX SuperPOD
Nodes	636	980	560
Processors (per node)	2 × 24 cores Intel Xeon Platinum 8260 @ 2.4 GHz	2 × 16 cores IBM POWER9 AC922 @ 2.6(3.1) GHz	2 × 64 cores AMD EPYC 7742 @ 2.25(3.4) GHz
GPUs (per node)		4 × NVIDIA Volta V100 SXM2 (2 pairs), 16 GB HBM2	8 × NVIDIA Ampere A100 SXM4 (with NVSwitch), 80 GB HBM2e
Cores	48 cores/node	32 cores/node, Hyperthreading x4	128 cores/node
RAM	384 GB/node	256 GB/node	1 TB/node
Node Performance (peak)	3.53 TFLOPS	32 TFLOPS	82.2 TFLOPS
Network Cards	Mellanox Infiniband 100GbE	1 × Mellanox ConnectX-4 EDR (100 Gb/s)	8 × Mellanox ConnectX-6 HDR (200 Gb/s)
Network Topology	Full Fat-tree	DragonFly++	Full Fat-tree

The speed-up *S* for an arbitrary number of ranks *n*, with respect to a reference minimum number of ranks *n*^min^, is defined as
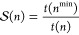
1where *t*(*n*) and *t*(*n*^min^) are wall times, whereas the parallel efficiency ε is
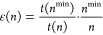
2The latter is a number in the interval [0,1], where ε = 1 is the ideal efficiency corresponding to a perfect speed-up in which *t*(*n*) is exactly *t*(*n*^min^)/*n*.

### 3.1 Ground State Energy Calculations for Large Systems

In order to show the performance of the core code of the Quantum ESPRESSO suite, PWscf, we present here calculations on a large orthorhombic supercell of chromium iodide (CrI_3_) bulk, with 1152 atoms, 7776 electrons, and cell parameters *a* = 22.48 au, *b*/*a* = 1.67, and *c*/*a* = 13.86. This calculation is part of a larger study of edge magnons,^43−45^ where interfaces between different crystalline phases are tested within this large supercell. The LSDA method with collinear spin polarization has been used, in combination with norm-conserving pseudopotentials generated with the atomic code,^46^ with plane-wave kinetic energy cutoff of 60 Ry. A Gaussian smearing with broadening of 0.01 eV was also used, resulting in a total number of 4666 Kohn–Sham states. A uniform Monkhorst–Pack^29^ grid of 4 × 1 × 1 **k**-points has been used, resulting into 3 **k**-points per spin component.

**Figure 5:**
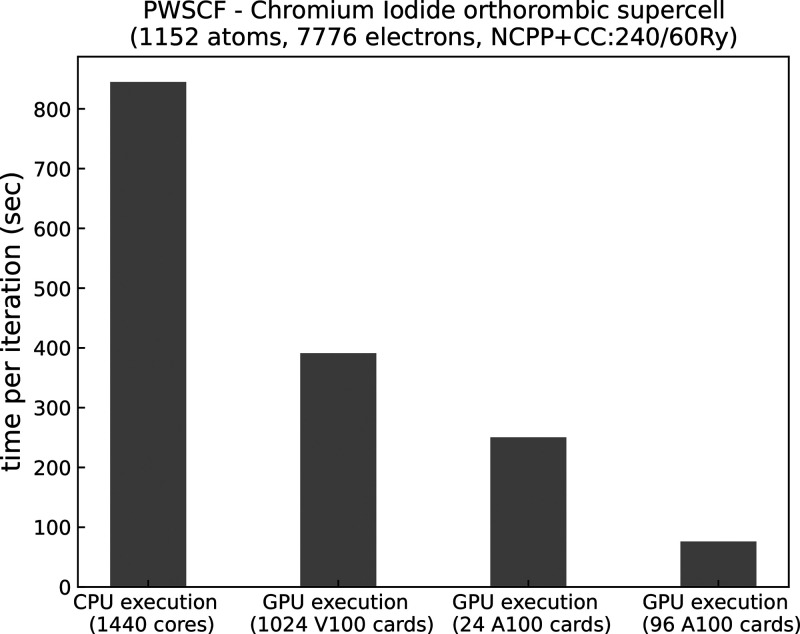
Time per SCF iteration of a chromium iodide (CrI_3_) orthorhombic supercell (*a* = 11.89 Å, *b* = 19.81 Å, *c* = 164.81 Å), norm-conserving pseudopotential with core correction, cutoffs 60 and 240 Ry for wave functions and density, respectively. Each SCF iteration corresponds to a Davidson diagonalization.

In Figure 5 the time per iteration of CPU execution on Galileo100 and GPU execution on Marconi100 and Selene supercomputers is reported. In each case we picked the best results in terms of balance between performance and memory consumption, obtained after running several calculations with different computational configurations. For CPU calculations on Galileo100, the setup with 30 nodes (1440 cores) allows an equally distributed workload of 3 **k**-points and two spin channels among 2, 3, or 6 *pools*. Using fewer nodes, either performance was inferior or there was not enough memory to fully test *pool* parallelism. On the other side, calculations with 32 nodes were affected by the maximum FFT dimension: there are 1536 FFT grid points along the longest cell dimension (*c* = 164.81 Å) and exactly 1536 cores, leading to too few plane waves per rank.

**Figure 6:**
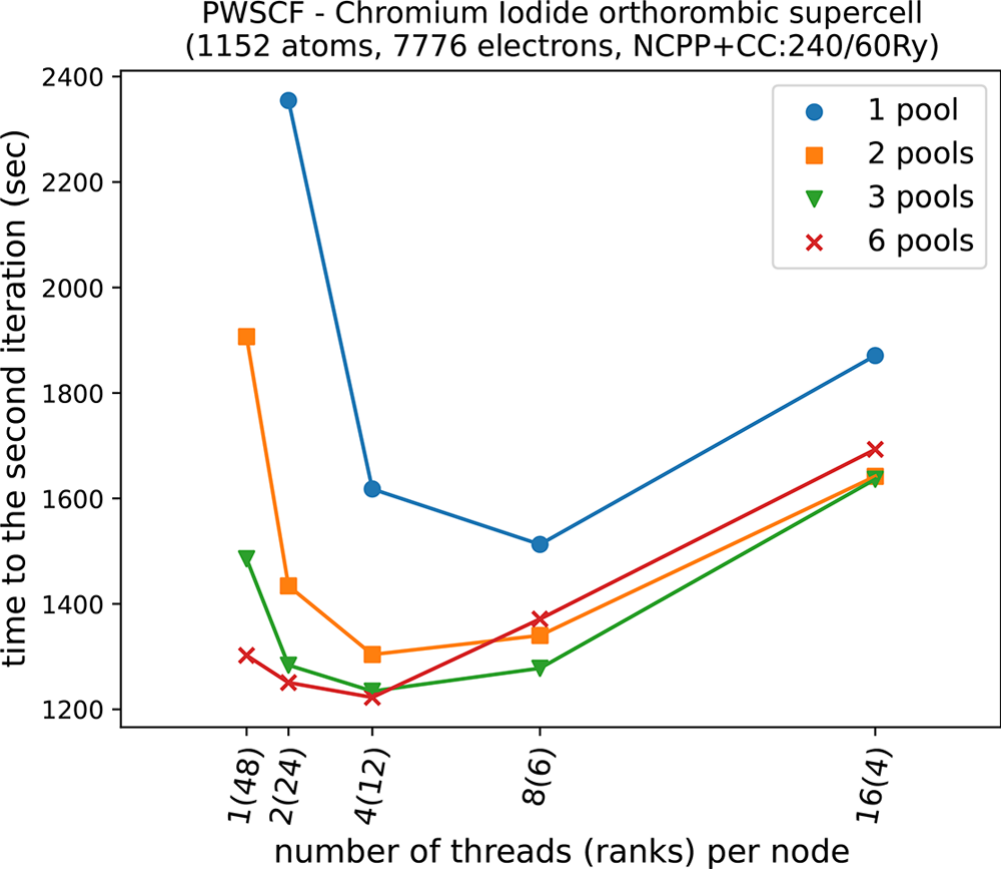
Time to the second SCF iteration (initialization + first iteration) of a chromium iodide (CrI_3_) orthorhombic supercell (*a* = 11.89 Å, *b* = 19.81 Å, *c* = 164.81 Å), norm-conserving pseudopotential with core correction, cutoffs 60 and 240 Ry for wave functions and density, respectively. Different numbers of *pools* and different threads/ranks combinations are compared at a fixed number of 1440 total cores (30 nodes).

We also tested different combinations of *pools*, MPI ranks, and OMP threads, reported in Figure 6. Increasing the number of *pools* markedly improves computational times because calculations for different **k**-points are decoupled (cf. also Figure 4). When using extreme MPI parallelizations (1 thread per node, i.e., 1440 ranks), communications among ranks become predominant, whereas using too many threads leads to too small *pool* groups, and loops over **G**-vectors become more demanding. These two competitive trends lead to the nonmonotonic curves with minima shown in Figure 6, also often found in other benchmarks of the Quantum ESPRESSO codes (as for example here^47^). Optimal combinations found here are with 2 or 4 threads and 3 or 6 *pools*. Data in Figure 5 refer to 2 OMP threads per rank and 6 *pools* of 5 nodes (120 ranks) each.

On Marconi100, calculations with 16, 32, 64, 80, and 128 nodes were limited by memory requirements even with only one *pool* (16 GB is the maximum available for V100 GPU), and the time reported in Figure 5 refers to 256 nodes with *R&G* parallelism only. Eight OMP threads per rank have been used to fully deploy the CPU cores of the nodes. Despite the limitations, running on V100 GPU cards is significantly faster than running on CPU leveraging *pool* parallelism.

The benchmark executed on Selene overcomes the memory limitations of the V100 cards thanks to 80 GB of GPU memory. In fact, bigger GPU memory capacity allows us to run the full calculation with only 24 GPUs (with 16 OMP threads, no *pools*), showing an overall speedup with respect to the reference CPU execution of about 4×. It is also possible to fully exploit *pool* parallelism by increasing the number of nodes to avoid memory capacity constraints, further improving performance. For example running on 96 A100 GPUs with 3 *pools*, the time per iteration drops down from 250 to 76 s, resulting in a speed-up of about 10× with respect to the CPU reference calculation and a dramatically smaller amount of resources employed.

Generally speaking, for PWscf and most other codes in the Quantum ESPRESSO suite, GPU memory is a quite crucial parameter. A large GPU memory allows both reduction of the total number of ranks employed and an increase of the number of *pools*, ultimately reducing communications and host–device synchronizations. On GPU architectures with limited memory, instead, calculations for large molecular systems may require an exceedingly large number of nodes.

### 3.2 Phonons and Vibrational Properties via DFPT Methods

The entire PHonon code^7,8^ has been accelerated using the approach described in Section 2, based on OpenACC and relying on CUDA Fortran modules and libraries inherited from PWscf.

**Figure 7:**
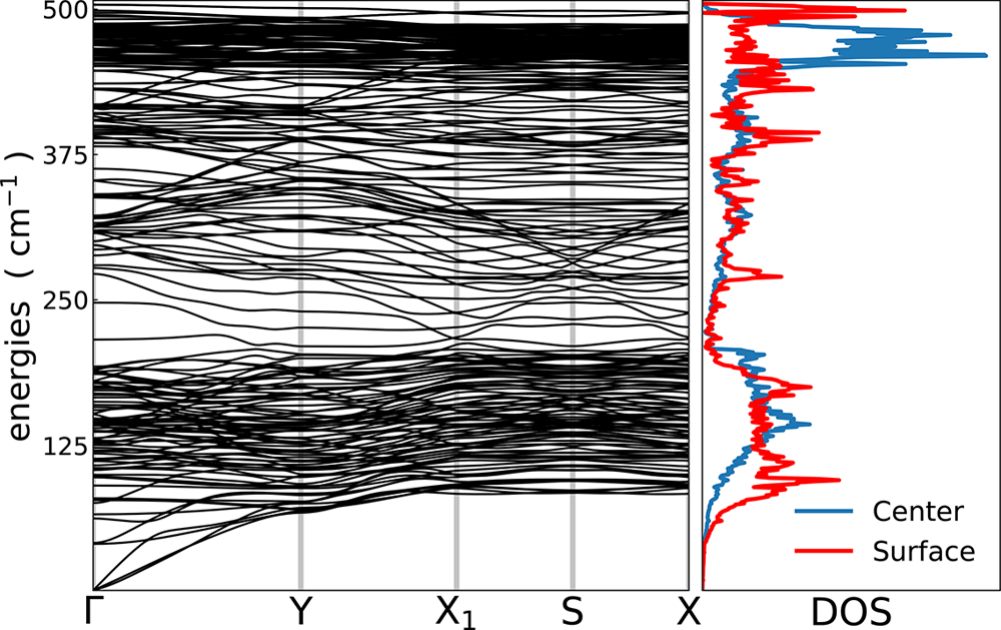
Phonon dispersion and projected density of states of the silicon (100) surface simulated using the Si(100)-16L model.

In Figure 7 we show the phonon dispersions of the 100 surface of silicon with c(4 × 2) reconstruction (reported among the most stable ones by a number of previous studies^48,49^), simulated using a base centered orthorhombic primitive cell (*a* = 2*b*).^48,50^ The first Brillouin Zone of the slab has been sampled with a 8 × 8 × 1 uniform Monkhorst–Pack mesh^29^ (21 total symmetry inequivalent **k**-points), whereas two different depths have been considered in the third direction, one with 16 layers of silicon atoms (referred to as Si(100)-16L hereafter, with 64 atoms per cell) and one with 32 layers (Si(100)-32L, 128 atoms per cell).

The phonon dispersions have been obtained using a standard procedure based on Fourier interpolation technique, evaluating the dynamical matrices in reciprocal space on a uniform 8 × 8 × 1 grid of **q**-points, and then interpolating along the path Γ-Y-X_1_-S-X, used in another previous work.^51^ PBE^52^ functional and norm-conserving pseudopotentials^53^ with a plane-wave kinetic-energy cutoff of 60 Ry have been employed. We observe that the depth of the slab is large enough to recover the periodic structure of the bulk phonons, resulting in a realistic description of the physical surface of the material, even with the smaller model with 16 layers.

The computationally most intensive part of the dispersion calculation is the evaluation of the dynamical matrices in reciprocal space for all of the necessary **q**-points. For each **q**-point, orbitals and band energies at all (**k** + **q**)-points need to be computed with a non-SCF calculation, and a set of Sternheimer equations^42^ has to be solved for each symmetry-inequivalent perturbation. In the present model systems, the perturbations are 192 and 381 irreducible representations of nuclear displacements (*irreps* hereafter), for Si(100)-16L and Si(100)-32L, respectively. In the PHonon code, this heavy computational burden can be distributed by using the parallelization schemes briefly sketched in Section 2.6.

*Images* can be used to create nearly embarrassingly parallel tasks, each devoted to the computation of dynamical matrices for a subset of **q**-points and *irreps*. However, perturbations with **q** ≠ 0 lower the symmetry of the system and require a number of symmetry-independent (**k** + **q**)-points that depend upon the **q**-point. As a consequence, the workload of different *images* can be quite unbalanced, and the efficiency of the overall distribution pattern depends on the particular system. For example, the present calculation has been distributed over 144 MPI ranks, subdivided in 9 *image* groups, with total number of (**k** + **q**)-points varying from 21, 68, 72 and 128.

**Figure 8:**
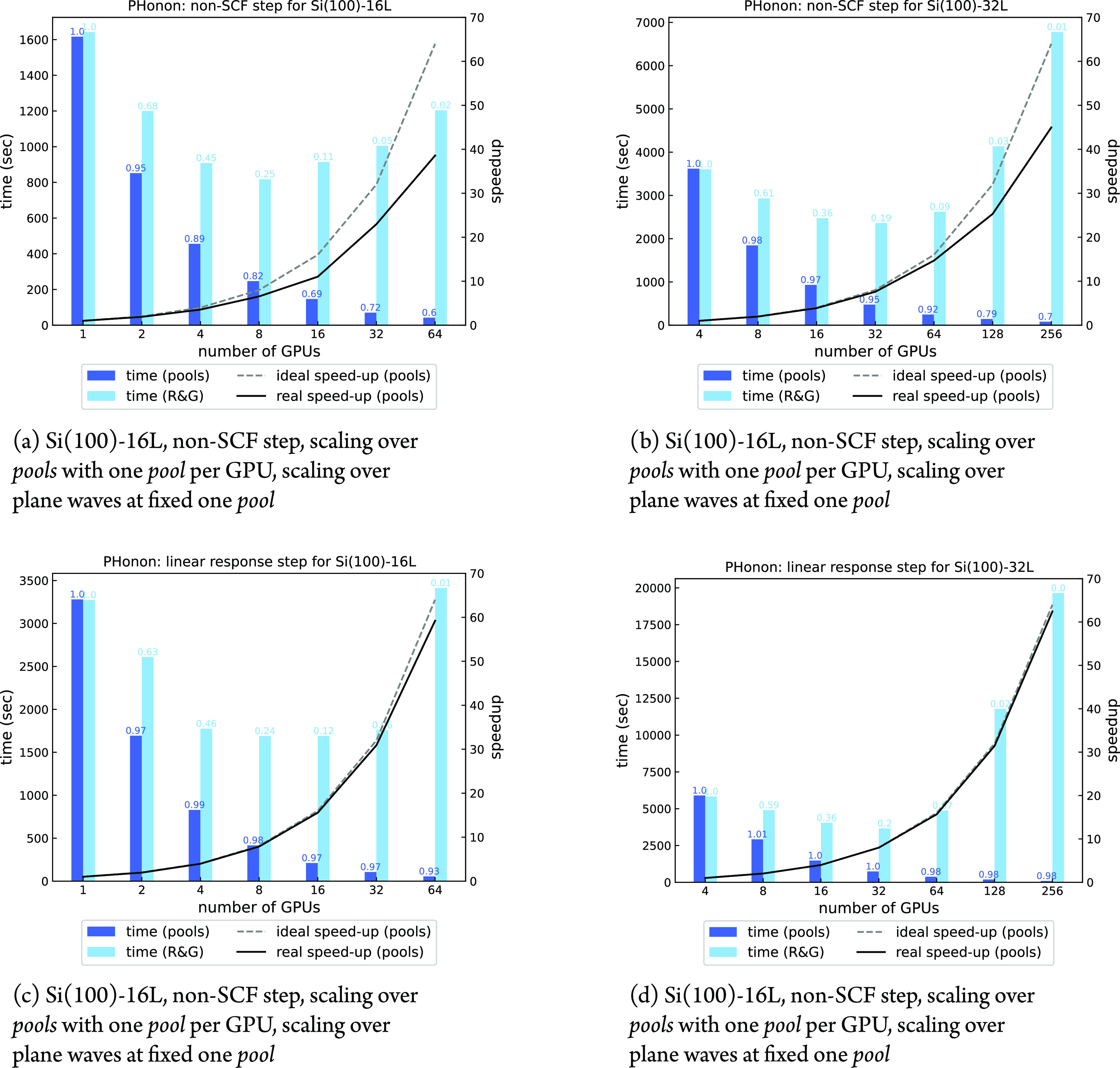
Scaling over *pools* and plane waves for one irreducible representation of a phonon calculation at fixed , with 128 (**k** + **q**)-points. All calculations were done using one *image* and 8 OMP threads. Computational efficiency is reported on top of each bar.

Let us first analyze the performances inside each *image* group and analyze how to optimally distribute the available ranks among *pools* and *R&G* groups. Figure 8 panels a–d show the scaling performance over *pools* and plane waves of the most computationally expensive non-SCF and Sternheimer steps (with 128 (**k** + **q**)-points) for one single *irrep* at a fixed **q**-point. The maximum number of usable *pools* is 64, considering that each *pool* must have at least two points, namely, **k** and **k** + **q**. The *R&G* parallelism has an upper bound in the number of planes along the *z*-direction of the FFT grid: 256 and 512 for the Si(100)-16L and Si(100)-32L respectively. An arbitrary number of 8 and 2 OMP threads has been chosen for GPU and CPU executions, respectively, on the basis of single-node measurements (not reported here) and empirical observations reported in Section 2.6.

In all cases, we observe very good efficiencies and speed-ups when scaling over *pools*, while using *R&G* parallelism the efficiency tends to saturate earlier, with a lower amount of resources employed. Of course, the price paid for the high efficiency of the *pool* parallelism is a less favorable memory allocation, which is especially important when running on accelerated architectures. For example, the runs over 256 GPUs shown in Figure 8b,d allocated about 13 GB of GPU memory when distributed over *pools* and less than 5 GB when distributed over plane waves.

**Figure 9:**
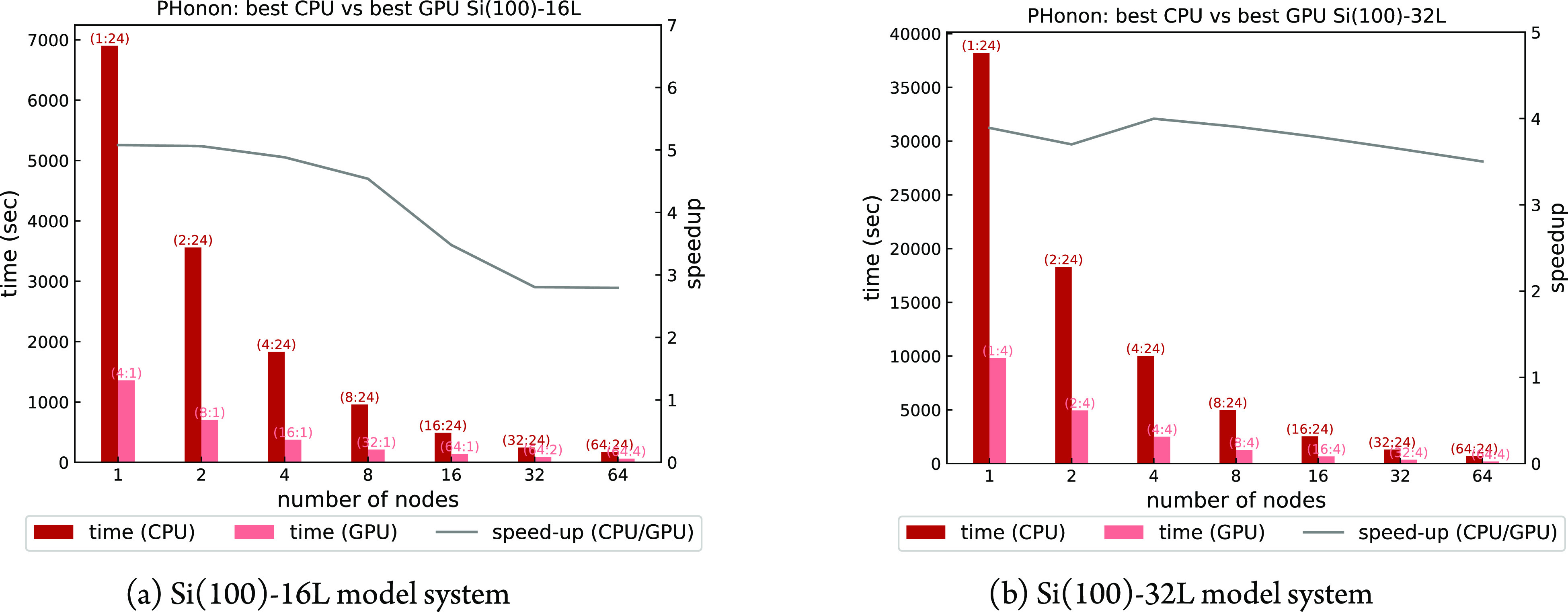
Time to solution and CPU/GPU speed-up of a phonon calculation at fixed , with 128 (**k** + **q**)-points and one irreducible representation, using one *image* and different combinations of *pools* and *R&G* groups (*pools*:*R&G*). GPU and CPU calculations have been performed with 8 and 2 OMP threads, respectively, to optimize the respective performance.

In Figure 9a,b a node-based performance comparison between our “best” CPU and GPU executions is shown, from 1 (48 cores versus 4 GPUs and 32 physical cores) to 64 nodes (3072 cores versus 256 GPUs and 128 physical cores); the latter is the maximum number of available nodes for production. The “best” CPU executions have been done using one *pool*, 24 MPI ranks per node and 2 threads per rank, and have been chosen among a set of single-node tests performed using different combinations of 1 and 2 *pools* per node and 1, 2, and 3 OpenMP threads per rank.

Regarding the GPU execution, for the smaller Si(100)-16L model, the optimal runs are those with one *pool* per GPU with no *R&G* parallelism—i.e., up to 16(64) nodes(GPUs)—that minimize communications among GPUs. Above 16(64) nodes(GPUs), the performance decreases to 3× due to the use of the additional plane-wave groups needed when *pool* parallelism is saturated.

For the larger Si(100)-32L model, the number of plane waves does not fit into one GPU memory. The best performances are obtained with one *pool* per node, with an *R&G* distribution of 4 ranks per *pool*. The maximum CPU/GPU speed-up achieved is around 5 for the smaller system and 4 for the larger system.

**Figure 10:**
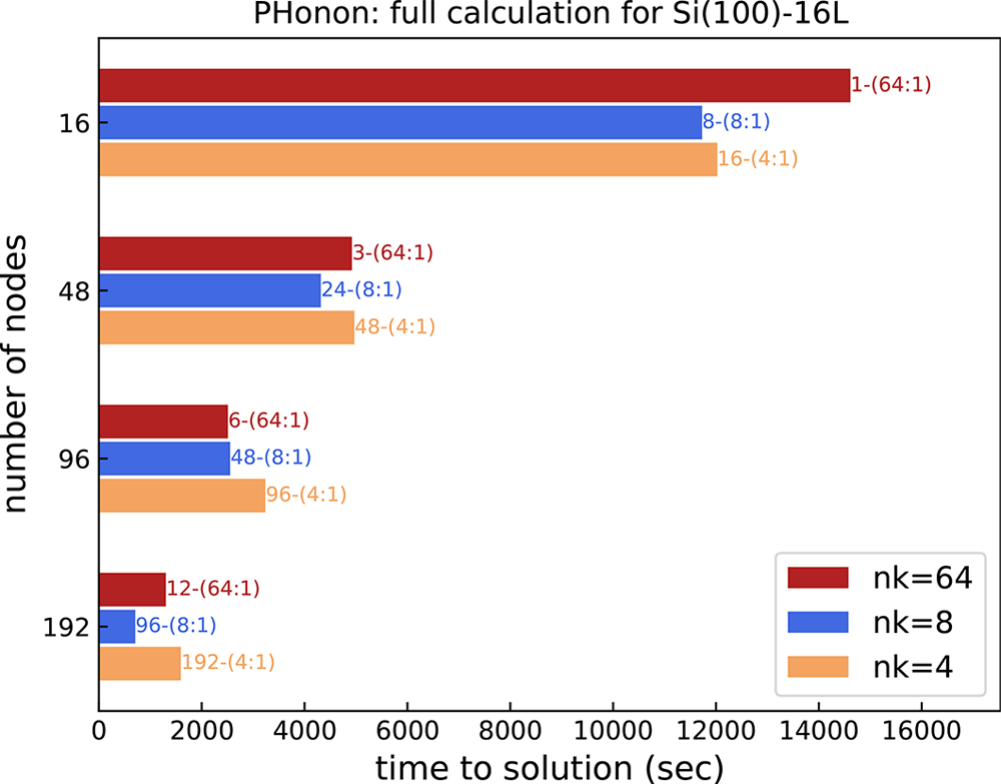
Time to solution (seconds) for one full phonon calculation at fixed , for all the 192 irreducible representations of the Si(100)-16L model system, 8 OMP threads, and different MPI parallelization schemes. The parallelization scheme is highlighted by the labels on top of the columns as *images*-(*pools*:*R&G*).

Once we now know how to configure parallelism inside a single *image*, we can move forward analyzing parallelism on multiple *images*. Figure 10 displays the times to solution to compute one full dynamical matrix on GPU, at one fixed **q**-point, including all the *irreps* of Si(100)-16L. Based on the previous discussion, *R&G* parallelism has not been used, and only *images* and *pools* have been varied, at a fixed *pool* size of one. Besides the obvious consideration that the calculations run faster on more nodes, an interesting aspect is that even at a fixed number of nodes, the choice of internal parallelism can play an important role for performance. This is especially evident for the most “extreme” computational setup, with 192 nodes, that shows variations in the computational times of more than 100% when changing the internal distribution of resources. If we transpose these variations in terms of GPU hours, the waste of resources due to a suboptimal choice of parallelism becomes even more dramatic. Noticeably, we observe that the best performance in Figure 10 is obtained when the available computational resources are balanced between *pools* and *images* (bars in the middle), whereas more “extreme” allocations, where one or the other parallelism is overloaded, tend to lead to a less favorable performance.

In summary, although it is very difficult to state *a priori* what is the best parallelization scheme for a generic system, we can draw some general guidelines from our benchmark study that are helpful in the choice of the parallelization scheme of PHonon calculations for accelerated architectures. First of all, *R&G* parallelism is less efficient than the other schemes for performance, but it can be very helpful for coping with memory limitations. Then, whenever possible, it is a good strategy to balance resource allocation among *pools* and *images* using the smallest possible number of *R&G* processes.

### 3.3 EELS Line Shapes via TD-DFPT Methods

The turboEELS code is used to simulate the electron energy loss (EELS) and the inelastic X-ray scattering spectra in periodic solids, using two methods based on the Liouville–Lanczos scheme^11,12^ and Sternheimer equations.^9,10^ Both methods have been recently ported to GPU using the approach described in Section 2.

**Figure 11:**
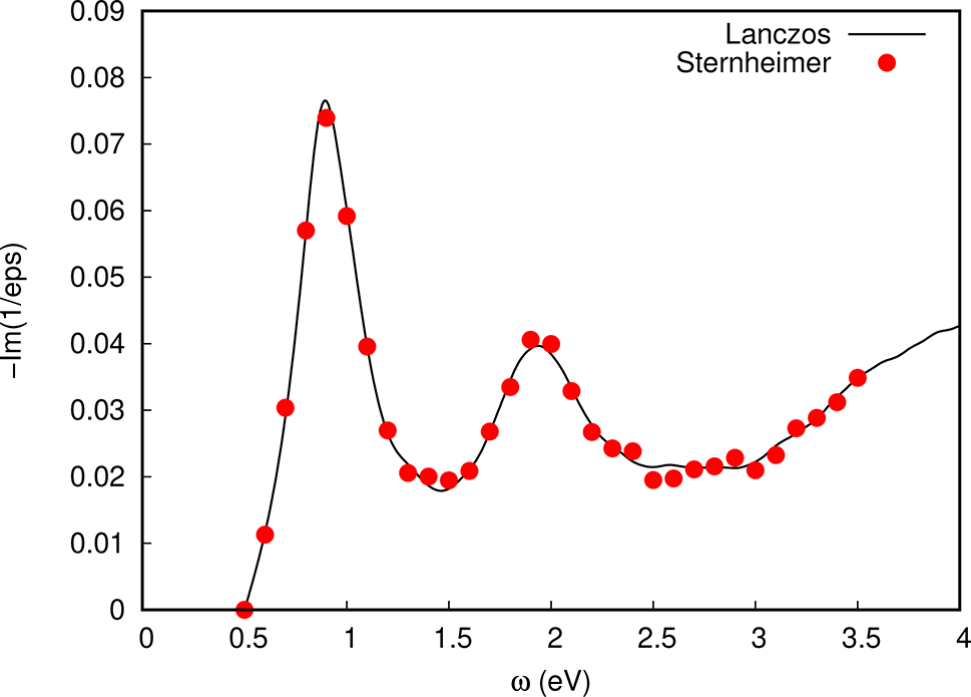
Electron energy loss spectrum of the silicon (100) surface, simulated using the Si(100)-16L model with transferred momentum |**q**| = 0.005 Ry along [011], a broadening of 0.01 Ry, and a scissor shift of +0.5 eV. The solid line shows the line shape calculated using the Lanczos algorithm (20k coefficient extracted), and red points refer to Sternheimer calculations.

In Figure 11 the contribution to the EELS spectrum arising from the imaginary part of the dielectric function is shown for the Si(100)-16L model system described in Section 3.2, using a transferred momentum of |**q**| = 0.005 Ry along the [011] direction. The spectral line shape has been computed using the Lanczos approach,^11,12^ with a Lorentzian broadening of η = 0.0035 Ry for the charge-density susceptibility (loss function) and 20000 iterations. Along the line shape, single spectral transitions computed with the Sternheimer method^9,10^ are shown, showing consistency between the two approaches. Noticeably, the spectral line shape is in fair agreement with the overall EELS spectrum obtained in more accurate and extensive studies.^48,49,54^

A turboEELS calculation with Lanczos scheme involves two main computationally intensive parts, namely, a non-SCF step, to compute wave functions and band energies at (**k** + **q**)-points, and a following Lanczos chains step. *Images* are not available here, and the calculation can be distributed by using *pools* and *R&G* parallelism only.

**Figure 12:**
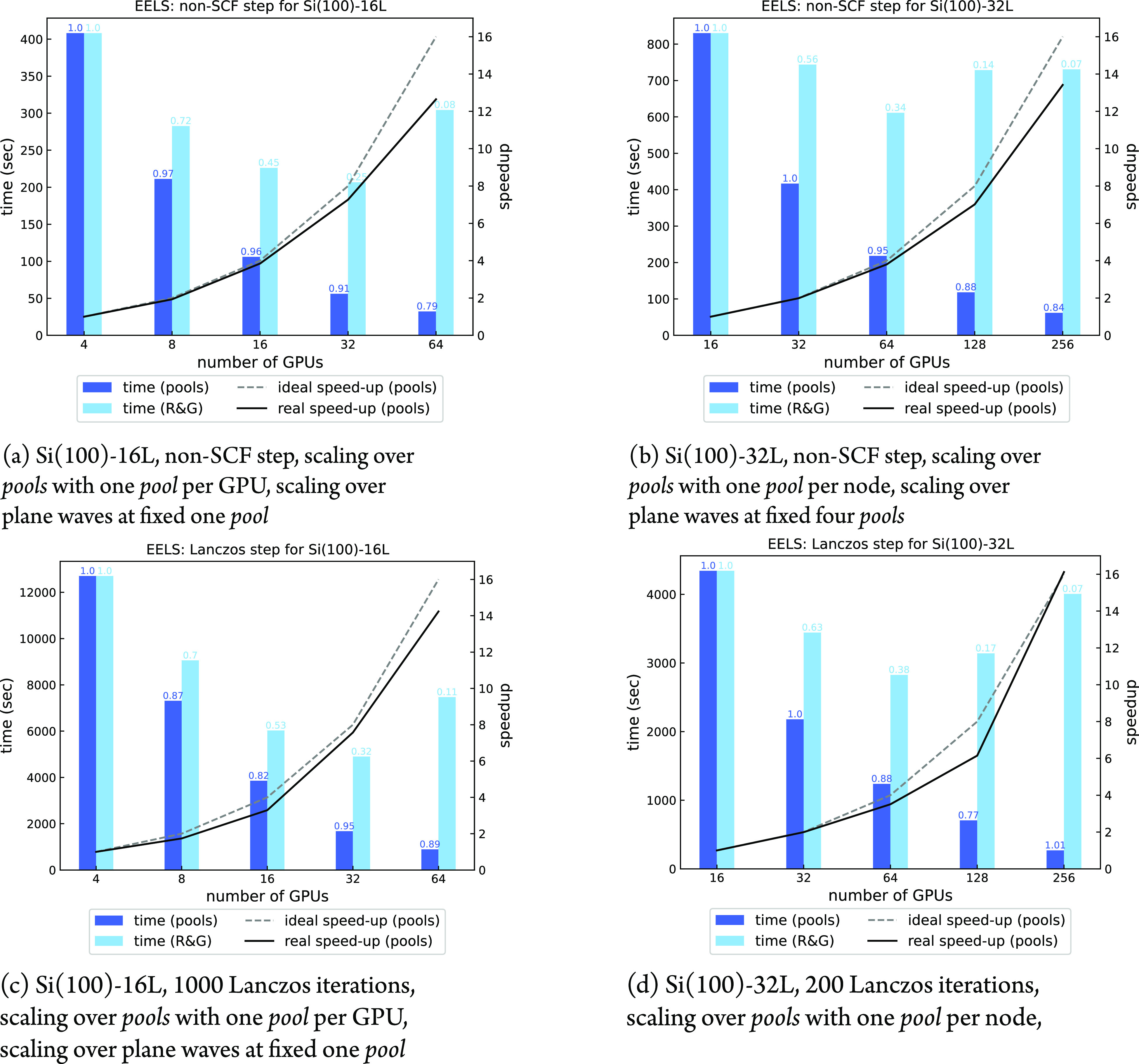
Scaling over *pools* and plane waves of different steps of an EELS calculation with transferred momentum **q**∥[011], |**q**| = 0.005 Ry, with 128 (**k** + **q**)-points. All calculations done using 8 OMP threads. Computational efficiency is reported on top of each bar.

In Figure 12a–d scaling over *pools* and plane waves of the two main steps of an EELS calculation are shown. Regarding the non-SCF step, we hereby note that, analogously to the phonon dispersion case discussed in the previous section, the chosen transferred momentum breaks all the crystal symmetries, resulting in a total number of 128 (**k** + **q**)-points (i.e., 64 **k**-points from the full 8 × 8 × 1 Monkhorst–Pack grid^29^ plus the equivalent (**k** + **q**)-point ones). The scaling of the non-SCF step for the EELS spectrum is thus fully comparable to the one discussed in Figure 8a,b, with similar speed-ups and efficiencies.

Also the speed-up of the Lanczos chain step is very good, especially for the larger system (Figure 12d), where it reaches the ideal value of 16 when 256 GPUs are used.

**Figure 13:**
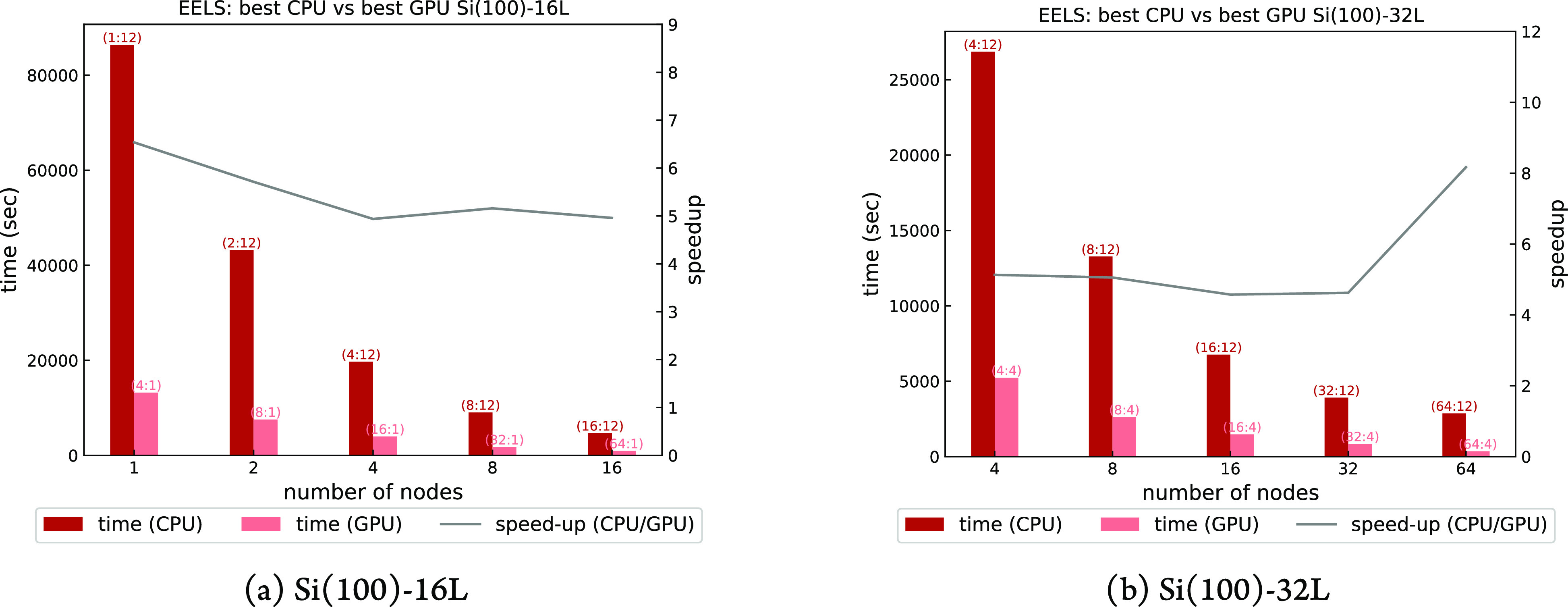
Time to solution and CPU/GPU speed-up of an EELS calculation with transferred momentum **q**∥[011], |**q**| = 0.005 Ry, with 128 (**k** + **q**)-points. All calculations done using 8 and 4 OMP threads for GPU and CPU executions, respectively.

In Figure 13a,b the node-based CPU/GPU comparison is shown, and we observe that the turboEELS code shows acceleration values of 5 to 6, comparable to the PHonon code. The “best” CPU executions have been done using one *pool*, 12 MPI ranks per node and 4 threads per rank, and have been chosen among a set of single-node tests performed using different combinations of 1 and 2 *pools* per node and 1, 2, 4, and 8 OpenMP threads per rank.

### 3.4 Time Evolution of Large Systems with Car–Parrinello Molecular Dynamics

For a detailed description of the Car–Parrinello (CP) method we refer to the original work.^16^

The Car–Parrinello code has been entirely ported to GPU following the approach described in Section 2, allowing the user to fully run a CP molecular dynamics simulation with nonzero initial wave function velocity on a machine with a GPU architecture.

**Figure 14:**
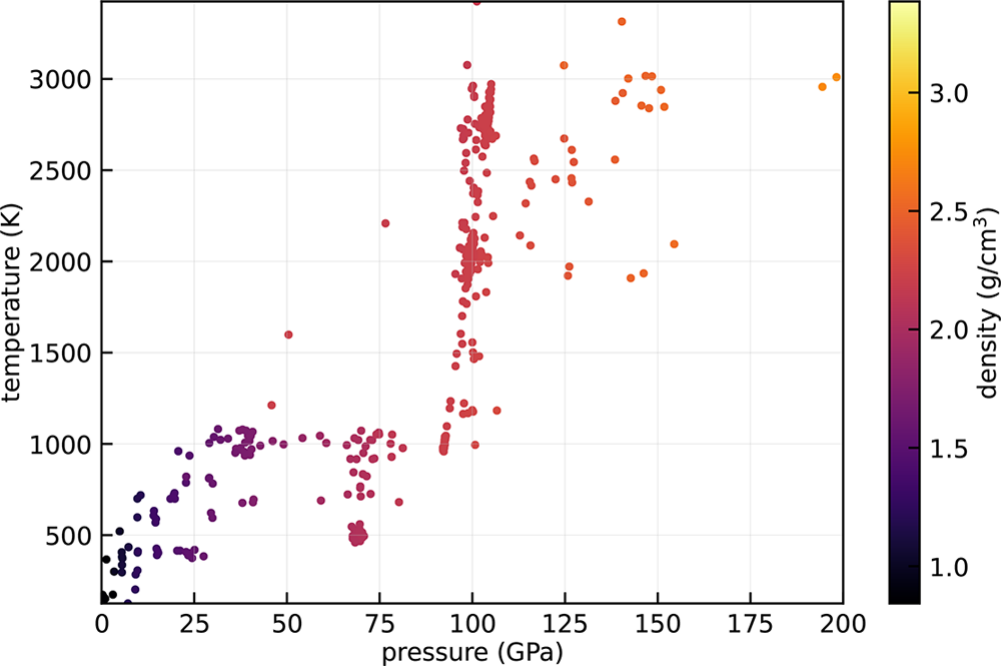
Equation of state diagram of ammonia, computed with the Car–Parrinello method. Each point is a CP run, and the color represents the density of the system for each particular combination of pressure and temperature.

The new code has been used to compute the superionic ammonia equation of state shown in Figure 14.^55^ The color map represents the density of the system, and its pressure and temperature are shown, respectively, on the *x* and *y* axes. The superionic ammonia has been modeled with a hexagonal close-packed cell of volume 1830 Å, with 144 nitrogen and 432 hydrogen atoms (1152 electrons). Norm-conserving pseudopotentials^53,56^ with nonlinear core correction have been used for both nitrogen and hydrogen atoms. The plane-wave cutoff was 90 Ry, sufficient for the full convergence of the energy, forces, and stress tensor. For the highest temperatures, near 3000 K, we used a time step of 0.041 fs, a fictitious electronic mass of 20 au, and a *emass_cutoff* of 2.5 Ry. At lower temperatures, we used larger time steps and electronic masses in order to speed up calculations. In all cases, we checked that a good enough conservation of the CP constant of motion was ensured.

**Figure 15:**
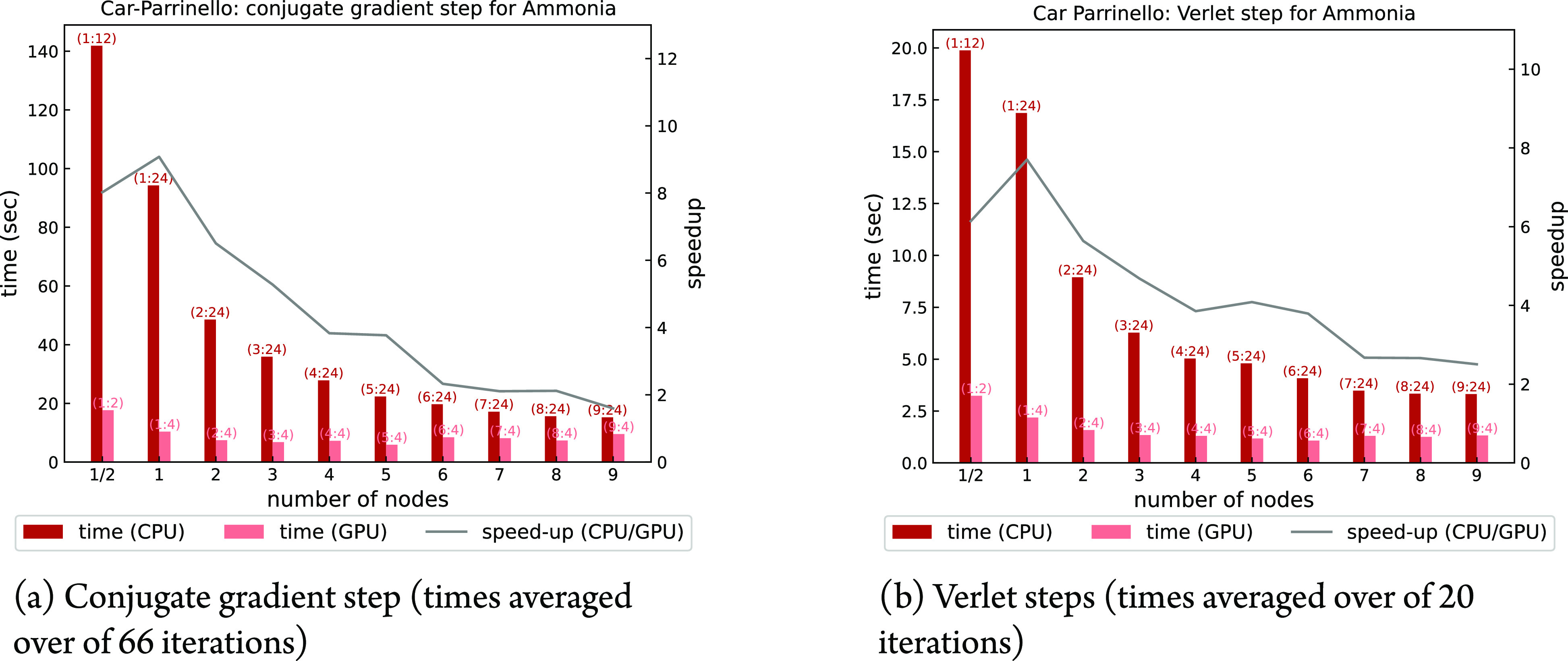
Times to solution and CPU/GPU speed-up of different steps of a CP simulation of Ammonia. CPU and GPU calculations done with 2 and 8 OMP threads, respectively.

In Figure 15, panels a and b show a node-based comparison between CPU and GPU scaling properties over plane waves of the conjugate gradient initialization and velocity Verlet step of a Car–Parrinello run on the ammonia system. Regarding CPU computations, we tried different computational setups with 1, 2, 4, and 8 threads per MPI process, and we choose those (2 threads) providing the best performance on Galileo100.

The optimal number of nodes for GPU calculations is 2, which is still significantly faster than calculations with 1 and a half nodes and negligibly slower than calculations with 3 nodes, outperforming roughly by a factor 2 the best CPU configuration with 9 nodes.

Also in this case, analogously to the PHonon and turboEELS codes, we found average speed-ups around 5–6 of GPU calculations with respect to the CPU ones.

## 4 Conclusions

In this article we have reviewed the current status of the Quantum ESPRESSO suite, with particular focus on the new developments done in the code since ref (2) was published.

We have first discussed the overall coding philosophy of the Quantum ESPRESSO project and of the GPU porting model, that has changed since the first versions of the code from a pure CUDA Fortran approach to a mixed interoperable OpenACC/CUDA Fortran scheme. The code refactoring done using the new scheme effectively allows people who are not necessarily experienced with GPU coding to keep contributing to the Quantum ESPRESSO project without the need to learn new languages. The porting experience described herein suggests that sometimes code developments done exclusively targeting performance might have downsides in terms of code readability and maintainability. It is thus very important to balance the effort to achieve outstanding performance gains with other relevant factors, such as simplicity of the code, ease of programming, and maintenance burden. In our case, the modularity of the codes also played a crucial role in the development process, allowing us to split the huge porting task into many smaller sub tasks and also allowing many different executables to take advantage of a relatively limited number of ported modules and libraries.

The GPU porting has significantly progressed in the last years, and now the most important codes of the suite, namely, PWscf, PHonon, turboEELS, turboLanczos, CP, and HP, are fully operative on heterogeneous architectures.

MPI data distribution and code parallelism schemes available in the Quantum ESPRESSO suite have been also reviewed in this article, highlighting the aspects related to GPU execution.

Extensive benchmark tests have been provided for the main codes to assess performance and identify best practices for launching calculations. Results depend to some extent on factors that are related to the algorithms used in the Quantum ESPRESSO codes and can be considered quite general. For example, when **k**-points are present, *pool* parallelism effectively allows strong scaling, whereas when massive resources are employed, it can be beneficial to exploit a fraction of the available cores as OpenMP threads, in order to reduce communication bottlenecks. However, performance is also strongly influenced by many other system-dependent factors, related to both the molecular system (e.g., symmetry, number of electrons) and the computational architecture (e.g., inter- and intranode communication bandwidths, number of RAM memory channels, CPU clock frequencies, GPU architecture, host-device communication bandwidths) that can affect latency and efficiency. With respect to the architectures employed here, computations on Marconi100 using V100 GPU cards are about four to six times faster than CPU computations using the same number of nodes on the Galileo100 cluster. Tests done using A100 cards on the Selene cluster also suggest even better performance on architectures based on more modern GPU technologies.

One of the main limitations found in the present version of the Quantum ESPRESSO codes is the difficulty to scale computations with no **k**-points, as *R&G* parallelism suffers from communication bottlenecks already at a relatively small number of ranks. In this respect, work is in progress to improve the current *band* parallelism and communication protocols in the MPI libraries of the Quantum ESPRESSO suite.
